# pH Stress Mediated Alteration in Protein Composition and Reduction in Cytotoxic Potential of *Gardnerella vaginalis* Membrane Vesicles

**DOI:** 10.3389/fmicb.2021.723909

**Published:** 2021-11-02

**Authors:** Parul Shishpal, Vainav Patel, Dipty Singh, Vikrant M. Bhor

**Affiliations:** ^1^Department of Molecular Immunology and Microbiology, Indian Council of Medical Research-National Institute for Research in Reproductive Health, Mumbai, India; ^2^Department of Biochemistry, Indian Council of Medical Research-National Institute for Research in Reproductive Health, Mumbai, India; ^3^Department of Neuroendocrinology and Transmission Electron Microscopy, Indian Council of Medical Research-National Institute for Research in Reproductive Health, Mumbai, India

**Keywords:** *Gardnerella vaginalis* membrane vesicles, pH stress, chaperones, flow cytometry, biogenesis, bacterial vaginosis, vaginal epithelial cells

## Abstract

The vagina of healthy women is predominantly colonized by lactobacilli but it also harbors a limited proportion of certain anaerobes such as *Gardnerella vaginalis*. An increase in *G. vaginalis* along with other anaerobes on account of perturbation in the vaginal microbiota is associated with bacterial vaginosis (BV). Although strategies adopted by *G. vaginalis* for survival and pathogenesis in a conducive environment (i.e., high vaginal pH, characteristic of BV) have been previously studied, the approaches potentially employed for adaptation to the low pH of the healthy vagina are unknown. In the present study, we investigated the effect of acidic stress on the modulation of the production and function of membrane vesicles (MVs) of *G. vaginalis*. pH stress led to a distortion of the bacterial cell morphology as well as an altered biogenesis of MVs, as revealed by transmission electron microscopy (TEM). Both qualitative and quantitative differences in protein content of MVs produced in response to pH stress were observed by flow cytometry. A significant change in the protein composition characterized by presence of chaperones despite a reduction in number of proteins was also noted in the stress induced MVs. Further, these changes were also reflected in the reduced cytotoxic potential toward vaginal epithelial cells. Although, these findings need to be validated in the *in vivo* settings, the modulation of *G. vaginalis* MV biogenesis, composition and function appears to reflect the exposure to acidic conditions prevailing in the host vaginal mileu in the absence of vaginal infection.

## Introduction

The mucosal lining of the vagina and the resident microbiota act as robust barriers against invading pathogens on account of the arsenal of antimicrobial substances such as defensins, mucins, and neutrophil gelatinase-associated lipocalin present in the cervicovaginal fluid (Aldunate et al., 2015).

Besides, the low pH (<4.5) maintained by the healthy vaginal microbiome comprising predominantly of *Lactobacillus* spp. also plays a key role in preventing urogenital infections including bacterial vaginosis (BV) which can lead to severe reproductive health complications (Reid, 2008; Witkin and Linhares, 2017). BV is characterized by a change in vaginal microbiome composition comprising of reduction in vaginal lactobacilli and a simultaneous increase in the abundance of anaerobic pathogens such as *Atopobium* spp*., Mobilincus* spp*., Streptococcus* spp., *Megasphaera* spp., *Gardnerella* spp., i.e., *G. vaginalis, G. leopoldii, G. piotii*, and *G. swidsinskii*, etc. (Jung et al., 2017; Vaneechoutte et al., 2019). *G. vaginalis* is a virulent, opportunistic microorganism commonly isolated from both symptomatic and asymptomatic women with BV. It has also been reported to be present in healthy women but at a lower abundance (2–7%) compared to women suffering with BV (11–29%; Shipitsyna et al., 2013; Ceccarani et al., 2019). Several studies have discussed the presence of different strains of *G. vaginalis* and their biofilm formation capacity under the conducive environment of high vaginal pH (Patterson et al., 2010; Castro et al., 2015). However, the precise manner in which *G. vaginalis* survives and adapts to the low vaginal pH has not been investigated in detail. This lack of information necessitates an investigation into the adaptive mechanisms employed by the bacterium to sustain under these stress conditions.

All bacteria are known to release membrane vesicles (MVs) which are required for intercellular communication and other vital functions. MVs are lipid-layered, non-replicative, nano-sized particles and have been reported in general to mediate various functions from bacterial defense to host pathogenesis (Avila-Calderón et al., 2014; Yu et al., 2017). Recently, we have reported the characterization of MVs produced by *G. vaginalis.* These MVs act as vehicles for virulence-associated factors and thereby contribute to host cell pathogenesis (Shishpal et al., 2020). However, it is not known whether the MVs produced by *G. vaginalis* play a role in adaptation and survival under stress conditions.

In view of the above, we aimed to study the effect of low pH exposure on the biogenesis, composition and function of *G. vaginalis* MVs. For this we employed, transmission electron microscopy (TEM) and dynamic light scattering (DLS) for determining morphology and size distribution of the MVs, proteomics for studying MV composition and flow cytometry for size based lipid and protein distribution within MVs. Further confocal microscopy and cytotoxicity assays were used for functional assessment of the MVs produced under acidic conditions.

## Materials and Methods

### Bacterial Culture and Growth Conditions

*Gardnerella vaginalis* ATCC 14019 (American Type Culture Collection, Manassas, VA, United States) was grown on Columbia agar base plates containing 5% human blood (Becton Dickinson) at 37°C for 48 h under anaerobic conditions using Anaerocult^®^ A (Merck Millipore, Germany) system in an anaerobic jar (Patterson et al., 2010). Cells were inoculated in brain heart infusion broth (BHI) supplemented with 2% (w/v) gelatin, 0.5% yeast extract, 0.1% starch, and 1% glucose (HiMedia, India), i.e., sBHI for 48 h at pH 6.5 (Rosca et al., 2020). 2 mL of 9.9M lactic acid (HiMedia, India) was added to sBHI medium (pH 6.5) to lower the pH of the medium to 3.5. Additionally, the cells were also transferred from sBHI medium (pH 6.5) to the same medium with pH 3.5 and allowed to grow for 48 h under conditions similar to that for pH 6.5. The cells cultured as mentioned above were used for all subsequent experiments.

### *Gardnerella vaginalis* Growth Curve

*Gardnerella vaginalis* was grown overnight in sBHI medium (pH 6.5) and further sub-cultured independently in the same medium, both at pH 6.5 and pH 3.5 under anaerobic conditions using Anaerocult^®^ A system (Merck Millipore, Germany) in an anaerobic jar as mentioned above. An equal volume of the culture was taken to measure the absorbance (OD_595 nm_) over time. Cells were pelleted at 4,000 × *g* for 10 min and stored at –20°C until preparation of whole cell lysates and determination of protein concentration and protein profile analysis by SDS-PAGE as described later. The growth curve analysis experiment including estimation of protein concentration and subsequent protein profile analysis was performed thrice. Also, cells collected at 48 h time point were plated on sheep blood agar plates (HiMedia, India) and incubated further under anaerobic conditions for 48 h at 37°C to determine the colony forming units (CFU). The CFU analysis was performed twice. The results were analyzed for statistical significance as mentioned in the section on statistical analysis.

### *Gardnerella vaginalis* Membrane Vesicles Isolation

*Gardnerella vaginalis* was cultured on Columbia agar base plates containing 5% human blood (Becton Dickinson), inoculated in sBHI medium and allowed to grow at pH 6.5 and pH 3.5, respectively, for 48 h. Then, the cell free supernatants (CFS) were filtered through 0.4 μm and 0.2 μm syringe filters (Merck Millipore) to remove cell debris. The filtrates thus obtained were subjected to ultracentrifugation at 100,000 × *g* for 3 h at 4°C to isolate MVs (Shishpal et al., 2020). Additionally, CFS (pH 6.5) was supplemented with 2 mL of 9.9M lactic acid to adjust the pH to 3.5 and was further incubated for 48 h followed by ultracentrifugation at 100,000 × *g* for 3 h at 4°C. The pellets were subsequently resuspended in phosphate buffered saline (PBS) at pH 7.4 (PBS), aliquoted and stored at –20°C till further processing. An aliquot of the MVs was used for determining the protein concentration by the Bradford reagent (Sigma) using BSA (0.1–1.4 mg/mL) as the protein standard as detailed in the subsequent section.

### Protein Profile Analysis of *G. vaginalis* Cells and Membrane Vesicles by SDS-PAGE

Stored bacterial pellets of *G. vaginalis* at pH 6.5 and pH 3.5 were subjected to whole cell lysate preparation with BugBuster reagent (BugBuster^®^ HT Protein Extraction Reagent, Novagen) using the manufacturer’s protocol. The protein concentration of the whole cell lysates of the bacterial pellets obtained for various time points during growth curve analysis was estimated by the Bradford assay (Sigma) with BSA (0.1–1.4 mg/mL) as the protein standard using a standard curve. To compare the protein profile of *G. vaginalis* cells at pH 6.5 and pH 3.5 over time, whole cell lysates were resolved using SDS-PAGE (10% resolving gel). Similarly, to compare the protein profile of *G. vaginalis* cells and MVs, an equal amount of protein (16 μg) from the whole cell lysate (collected at 48 h time point) and the *G. vaginalis* MVs were subjected to SDS-PAGE (10% resolving gel) at a constant voltage (100 volts) till the dye front reached the bottom of the gel, followed by staining of the gel with Coomassie brilliant blue R-250 (Sigma). SDS-PAGE analysis was repeated at least 5 times for both the whole cell lysates as well as MVs and qualitative changes in the protein profile were recorded.

### Transmission Electron Microscopy

To evaluate the effect of pH variation on bacterial cell morphology, TEM was carried out as previously described by Schooling and Beveridge (2006) with some modifications. 50 μL of bacterial cell suspension (30 mg/mL) was applied on formvar carbon-coated grids and allowed to dry at room temperature (RT) for 10 min. The coated grid containing the bacterial cells was stained with 2% uranyl acetate for 40 s, followed by washing with distilled water to remove excess stain and drying at RT. Micrographs were recorded using a Tecnai 12BT (FEI) transmission electron microscope at an acceleration voltage of 120 KV. TEM analysis was repeated at least three times for each of the conditions, i.e., growth of cells at pH 6.5 and pH 3.5. A total of 7–9 fields were observed per condition and the total number of MVs per field was determined.

### Dynamic Light Scattering

Dynamic Light Scattering measurements were performed to study the size distribution of MVs from *G. vaginalis* at the two different pH conditions. Particle size measurement was performed using Zetasizer Nano-ZS (Malvern Instruments, United Kingdom). The autocorrelation functions of the samples were analyzed using the Contin algorithm through the Zetasizer 7.11 software available with the instrument. Samples were run in triplicates.

### Flow Cytometry

To quantitate the size, protein and lipid distribution of MVs isolated under normal and acidic stress conditions, two different flow cytometers were used. Firstly, to detect the carboxyfluorescein succinimidyl ester (CFSE) stained MVs, a mixture of non-fluorescent beads of sizes 1, 2, and 4 μm (Invitrogen^TM^) were acquired with default threshold on FSC-A vs SSC-A scale using BD Accuri^TM^ C6 flow cytometer. The gating strategy included selection of a region corresponding to the region of beads of 1 μm as the MVs are expected to be approximately of a similar size. MVs were acquired at 20K threshold in R5 gate with a slow flow rate. To rule out non-specific staining with CFSE, BHI medium was processed in a manner analogous to the MVs. Further, the pellet of the BHI medium thus obtained as well as the supernatant remaining after isolation of MVs were used as controls.

As the BD Accuri^TM^ C6 flow cytometer was not found to be suitable for sub-micron analysis, it was used only for qualitative analysis. The quantitative analysis of MVs at submicron level was carried out using the BD FACSAria^TM^ Fusion flow cytometer. Prior to acquisition, MVs were stained with 10 μM CFSE (a protein staining dye) for 20 min at 37°C and 10 μg/mL of the lipid staining dye FM 4-64 FX (Invitrogen^TM^) for 10 min at 37°C (Pospichalova et al., 2015). The dual (i.e., both protein and lipid) stained populations were considered for quantitative analysis. CFSE has an excitation peak at 492 nm and emission at 517 nm and FM 4-64FX has excitation emission peak at 565 nm and 744 nm, being acquired in FITC and PE-Cy7 channels, respectively. A mixture of FITC labeled sub-micron particles (Invitrogen^TM^) of sizes 100 nm, 200 nm, 500 nm, and 1 μm were acquired on flow cytometry with a threshold of 100 on SSC, at slow flow rates and used to gate the dual stained MVs (Görgens et al., 2019). Similar parameters were used to acquire CFSE and FM 4-64FX stained MVs isolated under both pH conditions. Data analysis was carried out using FlowJo VX (TreeStar, Ashland, OR, United States).

### Mass Spectrometry and Data Analysis

To characterize the protein content of the *G. vaginalis* MVs at pH 3.5, nano LC-MS/MS (EASY-nLC 1000 system, Thermo Fisher Scientific) was performed using a methodology identical to that mentioned in our previous publication (Shishpal et al., 2020). Briefly protein sample (100 μg) from *G. vaginalis* MVs at pH 3.5 was treated with 6M guanidine-HCl followed by reduction with 5 mM Tris (2-Carboxyethyl) phosphine (TCEP), alkylation using 50 mM iodoacetamide and trypsin digestion. Trypsin digests were cleaned up using C18 silica cartridge as mentioned by manufacturer (The Nest Group, Southborough, MA, United States) and dried using speed vac. One microgram of the peptide mixture was resolved using 15 cm PicoFrit column (360 μm OD, 75 μm ID, and 10 μm tip) filled with 1.8 μm C18-resin (Dr. Maisch, Germany).

MS/MS scans were acquired at a resolution of 17,500 at m/z 400. The MS/MS spectra of peptides were analyzed using MaxQuant (version 1.5.3.8; with the Andromeda search engine). The peptides thus obtained were analyzed against the total predicted proteome of *G. vaginalis* for identification of proteins associated with the MVs. The protein false discovery rate was set to 1%. Subcellular localization of the proteome of *G. vaginalis* MVs obtained at pH 3.5 was compared with that of MVs obtained at pH 6.5 using UniProt repository^1^.

### Confocal Microscopy of Vaginal Epithelial Cells Treated With Membrane Vesicles

Confocal microscopy was used to study the effect of *G. vaginalis* MVs (obtained at both pH 6.5 and 3.5) on human vaginal epithelial cells (VK2/E6E7). Vaginal epithelial cells were grown in an eight-chamber slide (SPL Life Sciences, Korea) in keratinocyte serum-free medium supplemented with the bovine pituitary extract (50 μg/mL) and recombinant epidermal growth factor (0.1 ng/mL; Gibco, Invitrogen^TM^) at 37°C in a humidified atmosphere containing 5% CO_2_. Once the confluency reached 70–80%, cells were treated with CFSE stained MVs at a concentration of 300 μg/mL for 24 h, followed by washing with PBS and fixation with pre-chilled 3% formaldehyde for 10 min at RT. To observe changes in the cytoskeleton, cells in each well were stained with 250 μL of Phalloidin conjugated to BODIPY 558/568 dye (Invitrogen^TM^) at a dilution of 1:40 for 45 min at RT. Stained cells were washed twice with Dulbecco’s Phosphate Buffered Saline (DPBS) followed by nuclear staining with 250 μL of 1:2000 DAPI for each well and mounted with Vectashield mounting medium (Vector labs, United States). Imaging was done using a confocal laser scanning microscope-CLSM (Olympus Fluoview fv3000, Japan). Internalization of MVs within the vaginal epithelial cells was confirmed by serial Z section at intervals of 1 μm. All the experiments including treatment with MVs and imaging of the treated cells were carried out more than three times and all images thus obtained were studied for qualitative changes compared to untreated cells.

### Preparation of BSA Entrapped Liposomes

BSA entrapped liposomes were used as a negative control in experiments related to evaluation of the cytotoxicity of MVs as well as their potential to induce the production of the cytokine, IL-8 in vaginal epithelial cells, VK2. The liposomes were prepared by the thin film hydration method (Zhang, 2017). Briefly, 10 mg/mL phosphatidylcholine, was dissolved in 2:1 mixture of chloroform and methanol. Solvent was evaporated by using rotatory evaporator at 45°C to form a thin layer of lipid and left overnight at RT to evaporate the solvent completely. The dried thin lipid layer was resuspended in 10 mL of DPBS containing 10 mg/mL BSA followed by probe sonication at 30% amplitude for 5 min to obtain unilamellar vesicles.

### Cytotoxicity Assay and IL-8 ELISA for Vaginal Epithelial Cells Treated With Membrane Vesicles

VK2 cells were grown in 96 well plates and treated with increasing protein concentrations of MVs obtained at both pH 6.5 and pH 3.5 (9.37–300 μg/mL) and BSA entrapped liposomes (negative control) for 24 h at 37°C with 5% CO_2_. After completion of the incubation period, cell supernatants were removed and stored at –80°C for estimation of the levels of the cytokine, IL-8 using a commercially available sandwich ELISA kit (eBioscience, Thermo Fisher Scientific) according to manufacturer’s instructions. Cells were processed further by adding 10 μL of methylthiazolyldiphenyltetrazolium bromide (5 mg/mL) to each well, followed by incubation for 2 h in the dark. Following this, 100 μL of dimethyl sulfoxide was added to dissolve the formazan crystals and absorbance at 570 nm was measured. Untreated cells represented the 0% cytotoxicity control. Total lysis of cells following treatment with 1% (w/v) SDS represented the 100% cytotoxicity control.

### Statistical Analysis

All the data is representative of independent experiments and has been expressed as mean ± SD. The statistical significance of CFU and TEM analysis of *G. vaginalis* was determined using student’s unpaired *t*-test. Bacterial growth curve, protein estimation of the whole cell lysates, determination of percentage of MV sizes, median fluorescence intensity (MFI) of MVs at pH 6.5 and 3.5, cytotoxicity and ELISA were analyzed by two-way ANOVA with Bonferroni *post hoc* test and differences were considered significant at *p* < 0.05.

## Results

### pH Stress Reduces Growth of *G. vaginalis*

**FIGURE 1:**
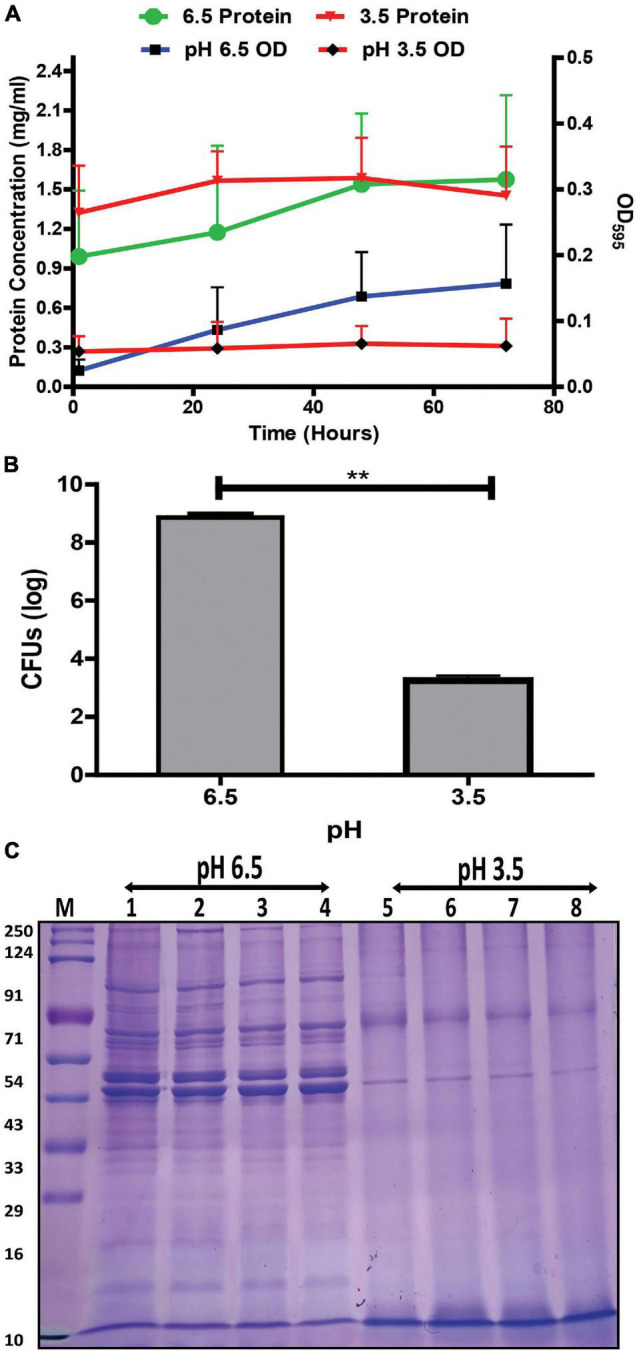
Effect of pH on the growth and protein profile of *G. vaginalis* ATCC 14019. **(A)** Changes in growth and protein concentration of *G. vaginalis* at pH 6.5 and 3.5 over time (1, 24, 48, and 72 h); data are presented as mean ± SD (*n* = 3). No statistical significance was observed upon analysis by two-way ANOVA with Bonferroni test. **(B)** Reduction in colony forming unit (CFUs) of *G. vaginalis* at pH 3.5 compared to pH 6.5 after 48 h; data are presented as mean ± SD (*n* = 2). Data was analyzed using student’s unpaired *t* test (**p < 0.01). **(C)** Time dependent (1, 24, 48, and 72 h) changes in protein profile of whole cell lysate of *G. vaginalis* at pH 6.5 and 3.5.

Effect of acidic stress on the growth kinetics of *G. vaginalis* was studied over time (1, 24, 48, and 72 h). The increase in absorbance of the *G. vaginalis* culture at pH 6.5 although not statistically significant, indicates a trend toward gradual increase in growth, over time while the absence of an apparent increase in absorbance suggesting no significant changes in growth was observed for bacteria grown at acidic conditions, i.e., pH 3.5 (Figure 1A) indicate pH stress mediated growth restriction. This was also supported by a significant, i.e., 2.7 log reduction (*p* < 0.01) in *G. vaginalis* cells numbers at pH 3.5 for the 48 h time point (Figure 1B). Despite a higher baseline protein concentration that was observed in the culture at pH 3.5 no significant change in protein concentration was observed over time. The apparent absence of changes in growth and protein concentration along with the reduction in viability of the *G. vaginalis* culture in response to pH stress was also reflected in the visible changes in the SDS-PAGE protein profile of *G. vaginalis* cells at pH 3.5 such as the reduction in both high and low molecular weight protein bands, reduced sharpness and intensity of the protein bands as well as apparent increase in the intensity of the lowermost protein band, i.e., approximately 10 kDa close to the dye front compared to that observed in cells grown at pH 6.5 (Figure 1C).

### Modulation in *G. vaginalis* Cells and Membrane Vesicles Under Acidic Stress

**FIGURE 2:**
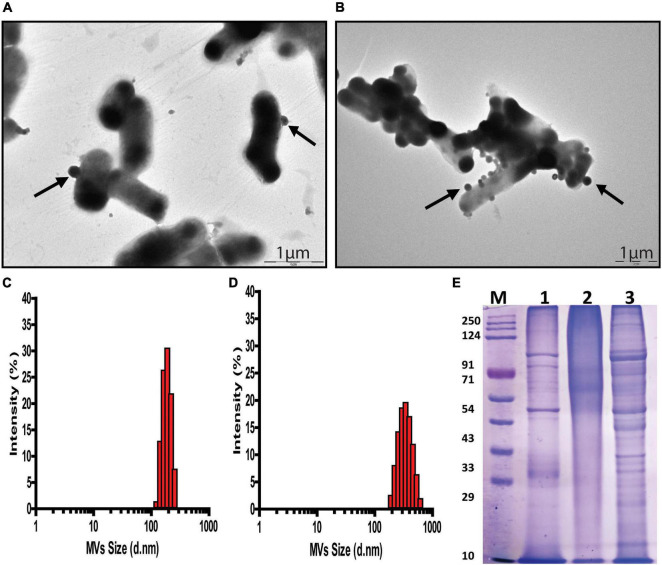
pH dependent changes in *G. vaginalis* ATCC 14019 membrane vesicles (MVs). Transmission Electron Microscopy (TEM) of negatively stained preparation of *G. vaginalis* cells showing released MVs attached to the cell surface (arrow) at pH 6.5 **(A)** and pH 3.5 **(B)**; scale bar-1 μm. Size distribution profile of MVs measured by dynamic light scattering indicates a diameter range of 190–250 nm at pH 6.5 **(C)** and 190–615 nm at pH 3.5 **(D)**. SDS-PAGE profile of *G. vaginalis* MVs isolated from (1) cells grown at pH 6.5, (2) cells grown at pH 3.5, and (3) cell free supernatant (cells grown at pH 6.5) supplemented with lactic acid to produce acidic conditions (pH 3.5) and (M) molecular weight marker **(E)**.

Transmission Electron Microscopy of *G. vaginalis* cells exposed to pH stress revealed an increase in budding of MVs (*p* = 0.015, Supplementary Figure 1) at the cell surface indicating an increase in MV production compared to those in cells grown at pH 6.5 (Figures 2A,B). Additionally, DLS showed a wider distribution as well as larger size of MVs from ∼190 to 615 nm at pH 3.5 compared to those at pH 6.5 (Figures 2C,D and Supplementary Tables 1 2). Apart from this, acidic stress also resulted in visible changes in the SDS-PAGE protein profile of MVs compared to MVs from cells grown at pH 6.5. Despite having equal protein concentrations as measured by Bradford assay, MVs produced at pH 3.5 displayed a diffused or smear pattern with absence of distinct protein bands in contrast to distinct and well separated protein bands of MVs obtained at pH 6.5. However, protein profile of MVs (pH 6.5 CFS) exposed to acidic conditions (pH 3.5) does not show any smearing pattern similar to that observed in MVs isolated at pH 3.5 (Figure 2E). Similar results were obtained in multiple replicates of the SDS-PAGE profiles of the MV proteins.

### pH Induced Changes in Morphology and Fluorescence Profiles of *G. vaginalis* Membrane Vesicles

**FIGURE 3:**
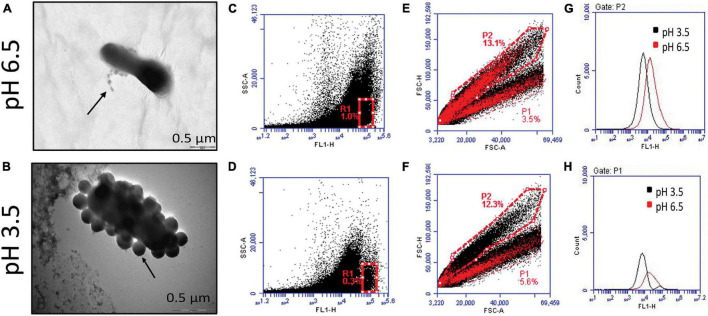
Characterization of *G. vaginalis* ATCC 14019 MVs obtained at pH 6.5 and 3.5 by flow cytometry. Enlarged images of negatively stained bacteria depicting release of MVs at pH 6.5 **(A)** and pH 3.5 **(B)**. Fluorescence based side scatter profile of MVs at pH 6.5 **(C)** and pH 3.5 **(D)** indicating the presence of a distinct subset at pH 3.5 gated as R1. Forward scatter plot of area vs height for total population of MVs at pH 6.5 **(E)** and pH 3.5 **(F)**, superimposed with R1 gated population. Fluorescence profiles of the P2 **(G)** and P1 subsets **(H)** of the total population **(E,F)** at pH 6.5 and pH 3.5.

As described previously, micrographs demonstrated the release of MVs at both pH 6.5 and pH 3.5. However, upon observation at a higher magnification, differences in cell morphology and the number of MVs released was apparent in cells exposed to acidic stress. The distortion in cell morphology arising on account of an apparent burst of vesicles at the cell surface observed under the stress condition was not seen at pH 6.5 (Figures 3A,B). In addition to this, flow cytometry-based characterization was carried out to determine changes in content of MVs. Non-fluorescent reference beads of 1, 2, and 4 μm were detected and gated based on their forward and side scatter profiles (Supplementary Figure 2A) and the region corresponding to beads of 1 μm were gated as R5 (Supplementary Figure 2B). Acquisition of CFSE labeled MVs, the supernatant obtained after ultracentrifugation as well as the pellet of the BHI medium obtained after ultracentrifugation was restricted to the R5 gate (Supplementary Figures 2C,D). Comparison of the fluorescence signals for all the above revealed a clear shift in the peak for MVs indicating a distinct fluorescence profile (Supplementary Figure 2E). Though, the overall fluorescence vs side scatter profiles (acquired in the R5 gate) of MVs obtained at pH 6.5 and 3.5 were similar, a distinct population was observed at pH 3.5 and was further gated as R1 (Figure 3D). A similar region was analyzed in case of MVs at pH 6.5 (Figure 3C). Doublet discrimination plots of forward scatter height vs area, typically used to identify singlets along the diagonal, were analyzed for the entire population of MVs (acquired in the R5 gate) at both pH 6.5 and pH 3.5. Superimposition of the R1 gated population in the area vs height plots represented in Figures 3E,F led to the identification of the portions of the total population which possessed higher fluorescence, i.e., MVs with an appreciable protein content. The P2 subset in MVs of pH 6.5 (Figure 3E) possesses higher fluorescence compared to that in MVs of pH 3.5 (Figure 3G) while the P1 subset in MVs both pH 6.5 and p H 3.5 possessed approximately equal fluorescence (Figure 3H).

### Quantitative Analysis of pH Induced Changes in *G. vaginalis* Membrane Vesicles Using Flow Cytometry

**FIGURE 4:**
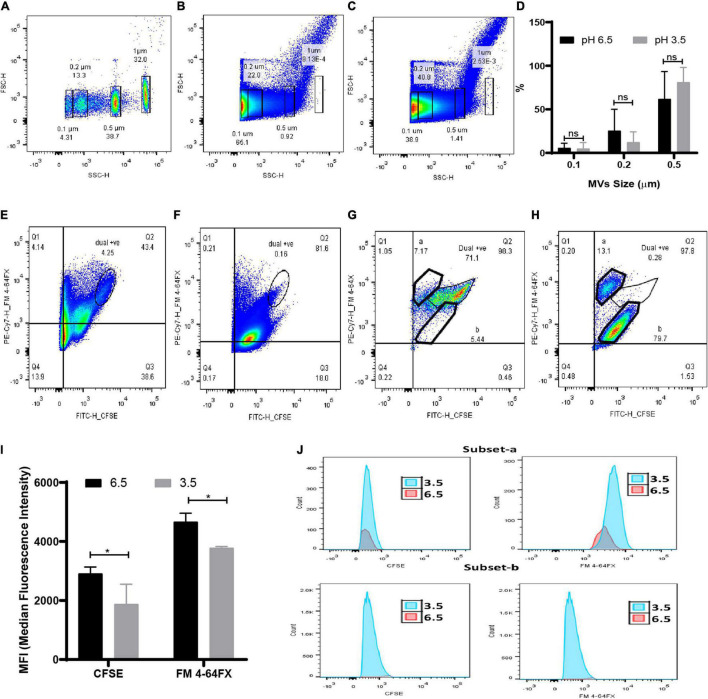
Quantitation of MVs at submicron level under acidic condition by flow cytometry. **(A)** Forward vs side scatter profile of FITC tagged sub-micron beads (0.1, 0.2, 0.5, and 1 μm). Representative scatter profile of protein (CFSE) and lipid stained (FM 4-64FX) MVs of **(B)** pH 6.5 and **(C)** pH 3.5 superimposed with reference beads gate. **(D)** Change in percentage of MVs of varying sizes at pH 6.5 and pH 3.5 corresponds to the superimposed gates on scatter plot of pH 6.5 and pH 3.5 MVs. Data are presented as mean ± SD (*n* = 3). Quadrant gate, Q2 represents dual positive population of 0.5 μm sized MVs of **(E,G)** pH 6.5 and **(F,H)** pH 3.5 and two sub population at **(G)** pH 6.5 and **(H)** pH 3.5. **(I)** Median Fluorescence intensity of dual positive population of CFSE and FM 4-64FX stained MVs (*p < 0.05). **(J)** Histograms of a and b subsets of pH 6.5 and 3.5 MVs.

Due to the limitations in obtaining a clear separation of sub-micron beads with the BD Accuri flow cytometer, quantification of MVs was carried out using the BD Fusion Aria flow cytometer. Since MVs consist of both lipids and proteins, quantitation of dual protein (CFSE) and lipid (FM 4-64FX) fluorescence was carried out to obtain a better estimate of the changes in *G. vaginalis* MVs brought about due to acidic stress. The distinct regions obtained in the side scatter vs fluorescence profile of the sub-micron beads (0.1, 0.2, 0.5, and 1 μm; Supplementary Figure 3) were used to demarcate corresponding regions in the forward vs side scatter profile of the bead mixture (Figure 4A). The strategy employed for the flow cytometry analysis is summarized in Supplementary Figure 4. In order to characterize the population of MVs of varying sizes that were obtained at pH 6.5 and pH 3.5, reference gates corresponding to beads of different sizes were applied on the forward vs side scatter profiles (Figures 4B,C). A decrease in the percentage of the total dual, i.e., CFSE and FM 4-64FX fluorescent population of MVs in the range of 0.2 μm (Supplementary Table 3) was observed at pH 3.5 along with an apparent increase in the percentage of the corresponding population of MVs in the range of 0.5 μm (Figures 4F,H). The changes in the percentage of the dual stained MVs observed at pH 3.5 indicate an alteration in the protein and lipid content due to changes in the distribution of protein across the population of lipid membrane bound vesicles, i.e., MVs, on account of the acidic stress (Figure 4D). In order to further investigate the apparent changes in protein and lipid distribution, we focused our attention on the MVs in the range of 0.5 μm owing to their higher protein fluorescence compared to those MVs in the range of 0.2 μm. Out of the entire dual fluorescence positive population represented in the Q2 quadrant, the region with maximum overlap of the protein and lipid fluorescence was gated as shown in fluorescence profiles for MVs around 0.5 μm obtained at both pH 6.5 (Figures 4E,G) and pH 3.5 (Figures 4F,H) from two different experiments. Although an apparent increase in total fluorescence of the dual positive population was observed at pH 3.5 (Figure 4D), analysis of the MFIs of the above mentioned regions revealed an overall reduction in protein as well as lipid fluorescence at pH 3.5 compared to that at pH 6.5 (Figure 4I). The reason for this apparent discrepancy is not known but the results clearly indicate that acidic stress leads to alteration in the fluorescence profile of the MVs. In case of the fluorescence profile for one experiment, the major dual fluorescence positive population observed at pH 6.5 was significantly diminished at pH 3.5 along with the appearance of two distinct subsets “a” and “b.” These subsets were not clearly evident at pH 6.5 (Figures 4G,H). Further, the MVs at pH 3.5 corresponding to subset “a” were lipid enriched while those corresponding to subset “b” were found to be protein enriched (Figure 4J). These results support the observation of uneven protein and lipid distribution in MVs (Figure 3) obtained at pH 3.5.

### pH Induced Changes in the Proteome of *G. vaginalis* Membrane Vesicles

**FIGURE 5:**
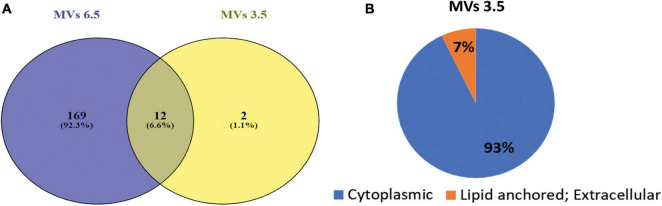
Proteomic analysis of *G. vaginalis* ATCC 14019 MVs in acidic stress. **(A)** Venn diagram demonstrating proteins common to MVs at pH 6.5 (Shishpal et al., 2020) and pH 3.5. **(B)** Subcellular localization of proteins of *G. vaginalis* MVs at pH 3.5 predicted using Uniprot and Locate P database.

Mass spectrometric characterization of the proteome of MVs obtained at pH 3.5 revealed a marked difference in protein composition along with a clear reduction in the total number of proteins compared to MVs obtained at pH 6.5 previously described in Shishpal et al. (2020). Compared to a total of 417 proteins identified in our previous study on MVs obtained from cells grown at pH 6.5, only 26 proteins (Table 1) could be identified in MVs at pH 3.5 in the present study. Out of these, 14 were found to have a peptide score more than or equal to 2 and twelve amongst these were also common to those found in MVs at pH 6.5 while one of the remaining two proteins was uncharacterized (Figure 5A). These proteins were categorized on the basis of predicted sub-cellular locations. Majority of the proteins (93%) were cytoplasm associated and the remaining (7%) were classified as lipid anchored and extracellular proteins by Locate P and Uniprot databases (Figure 5B). Functional annotation of the identified proteins indicated enrichment of cellular and metabolic processes such as carbohydrate metabolism.

**TABLE 1:** List of proteins identified in *G. vaginalis* MVs at pH 3.5.

Protein names	Accession no	Peptides	Function
Transaldolase	E3D9 × 8	19	Pentose-phosphate pathway
Elongation factor Ts (EF-Ts)	E3D9Z6	12	Translation
Probable phosphoketolase	E3D9B4	8	Unknown
DNA-binding protein HB1*	E3D9I0	4	Stabilization of DNA and preventing its denaturation under extreme environmental conditions
Glyceraldehyde-3-phosphate dehydrogenase, type I	E3D780	5	Glycolysis, moonlighting protein involved in colonization and invasion of host tissues
50S ribosomal protein L17	E3D7W2	2	Translation
Chaperone protein DnaK (HSP70)	E3D8E7	6	Maintains the assembly and disassembly of protein complexes, refolds the misfolded protein
Adenylate kinase (AK; Adenylate monophosphate kinase)	E3D7W7	3	Cellular energy homeostasis and adenine nucleotide metabolism.
30S ribosomal protein S6	E3D771	2	Translation
30S ribosomal protein S16	E3D753	4	Translation
2,3-bisphosphoglycerate-dependent phosphoglycerate mutase (BPG-dependent PGAM)	E3D976	2	Glycolysis, moonlighting protein involved in plasminogen binding
Uncharacterized protein*	E3D7I4	2	Unknown
50S ribosomal protein L11	E3D8P7	2	Translation
50S ribosomal protein L7/L12	E3D8P5	2	Translation
Phosphoglycerate kinase	E3D9V5	1	Glycolysis
50S ribosomal protein L9	E3D768	1	Translation
Ribosome-recycling factor (RRF)	E3D9Z8	1	Translation
Elongation factor P (EF-P)	E3D9U3	1	Translation
50S ribosomal protein L5	E3D7 × 6	1	Translation
Pyruvate kinase	E3DAG4	1	Glycolysis
60 kDa chaperonin (GroEL protein; Protein Cpn60)	E3D9J4	1	Prevents protein misfolding and promotes the refolding
Ribose-5-phosphate isomerase A	E3D8S4	1	Pentose phosphate pathway
ABC transporter, solute-binding protein	E3D881	1	Import of essential nutrients and export of toxic materials
Peptidyl-prolyl *cis*-trans isomerase	E3D8Z5	1	Protein Folding
Alpha-1,4 glucan phosphorylase	E3D7S8	1	Important allosteric enzyme in carbohydrate metabolism
Uncharacterized protein	E3D907	1	Unknown

*Proteins identified only in pH 3.5 MVs.

### Effect of pH Induced Changes in *G. vaginalis* Membrane Vesicles on Uptake by Vaginal Epithelial Cells

**FIGURE 6:**
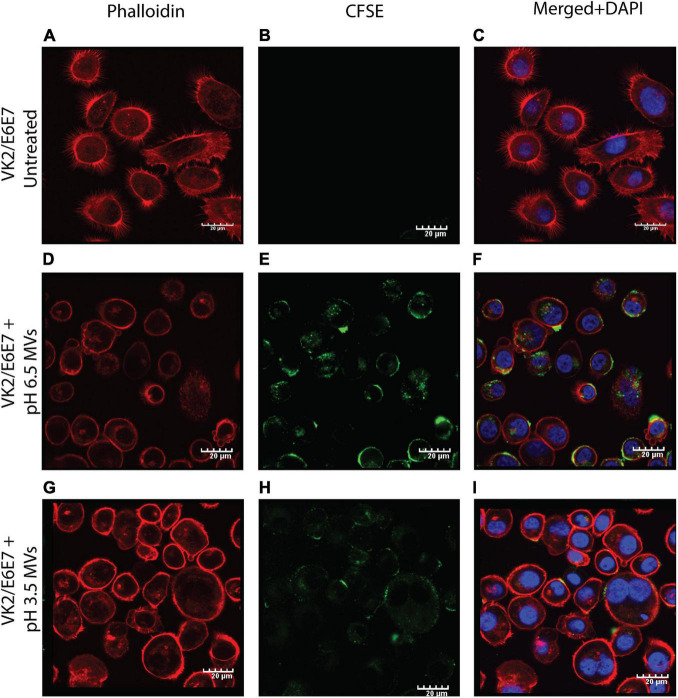
Adherence of *G. vaginalis* ATCC 14019 MVs and uptake by vaginal epithelial cells, VK2. Confocal microscopy sections of phalloidin (red) stained VK2/E6E7 cells treated for 24 h with CFSE (green) stained *G*. *vaginalis* MVs obtained at pH 6.5 **(D–F)** and pH 3.5 **(G–I)** along with untreated controls **(A–C)**. Nuclei were stained with DAPI (blue). Scale bar 20 μm.

The pH induced changes in *G. vaginalis* MVs (CFSE stained; green) appeared to have an effect on their adherence to and uptake by vaginal epithelial cells (VK2 cell line). Compared to pH 6.5 MVs which resulted in both adherence to and uptake by VK2 cells (Figures 6E,F), pH 3.5 MVs were found to only adhere to VK2 cells without evidence of any significant uptake (Figures 6G–I). Although blebbing and changes in actin cytoskeleton network (phalloidin staining; red) of the VK2 cells were observed upon treatment with MVs compared to untreated controls (Figures 6A–C), the extent of changes observed in case of pH 3.5 MVs suggests that the apparent reduction in fluorescence of the actin cytoskeleton (Figure 6D) was lesser than that seen with pH 6.5 MVs.

### Cytotoxicity and IL-8 Cytokine Induction by *G. vaginalis* Membrane Vesicles

**FIGURE 7:**
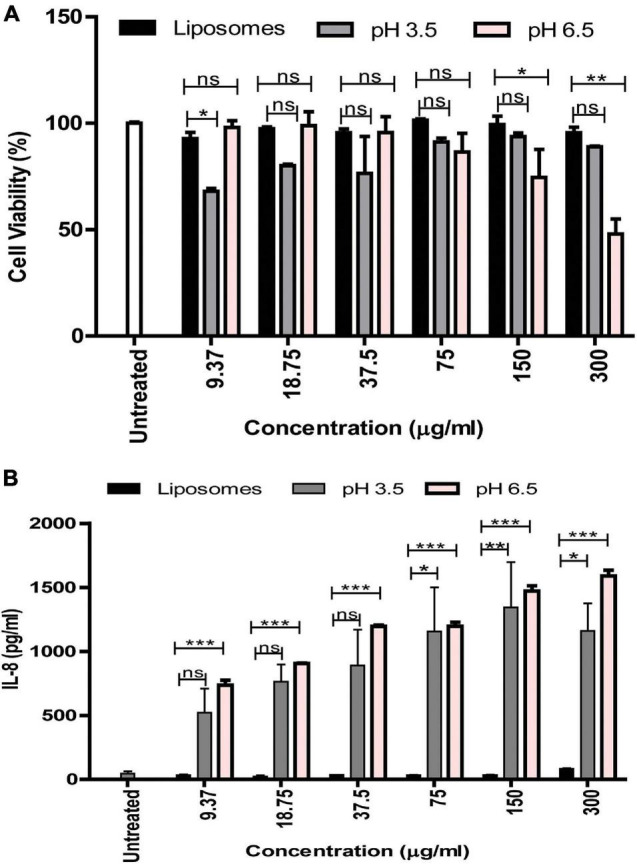
Effect of *G. vaginalis* MVs obtained at pH 6.5 and pH 3.5 on the viability and cytokine induction by vaginal epithelial cells. Cell viability **(A)** and induction of IL-8 **(B)** by VK2 cells in response to treatment with pH 6.5 and pH 3.5 MVs (9.37–300 μg/ml). BSA entrapped liposomes served as control and was used at protein concentrations similar to that of the MVs. Data are presented as mean ± SD. Statistical analysis was done using two-way ANOVA with Bonferroni test *p < 0.05; **p < 0.01; ***p < 0.001; and ns, non-significant.

In order to understand the functional relevance of the change in protein composition of MVs in response to acidic stress compared to MVs obtained at pH 6.5, their effect on viability of vaginal epithelial cells, VK2 was investigated. Although the pH 6.5 MVs resulted in a significant reduction in viability of the VK2 cells, pH 3.5 MVs were found to have no significant effect on the viability of VK2 cells with the exception of a reduction in viability at a protein concentration of 9.37 μg (Figure 7A). On the contrary, pH 3.5 MVs led to a significant dose-dependent increase in the levels of the pro-inflammatory cytokine IL-8 in the vaginal epithelial cells similar to that observed in case of pH 6.5 MVs (Figure 7B).

## Discussion

Bacteria are exposed to numerous environmental conditions including an alteration in physiological pH which may induce stress. In order to sustain under stress conditions, bacteria employ different strategies including the modulation of the cell membrane components, changes in macromolecules, synthesis of chaperones that are required to prevent protein and DNA damage, etc. (Haruta and Kanno, 2015). The emphasis of studies related to pH stress has been in the context of the microbiota of the gastrointestinal tract (Lund et al., 2014; Sanhueza et al., 2015). Compared to this, fewer studies have focused on stress response of the vaginal microbiota which is also exposed to low pH in case of healthy women. Further, the studies have been restricted only to lactobacilli (Witkin and Linhares, 2017) and there are no studies on mechanisms of adaptation to low pH by anaerobes, which may also inhabit the healthy vagina. In addition to the already mentioned approaches, secretion of MVs has also been proposed as a strategy for adaptation and survival under stress conditions (Volgers et al., 2018). MV formation is a ubiquitous process that occurs in both pathogenic and non-pathogenic organisms (Yu et al., 2017). This process has been widely characterized in physiological conditions as a defensive and offensive mechanism. This is on account of the fact that MVs transport virulence factors such as toxins, hydrolytic enzymes and other proteins which facilitate invasion, damage of host cells, down regulation of host immune responses, etc. MVs also carry components which degrade antibiotics, promote biofilm formation, etc. in addition to absorbing cell surface acting antimicrobial agents and protecting the bacterial cells. However, the contribution of MV production as a mechanism for adaptation to environmental stress has received less attention (MacDonald and Kuehna, 2013; Bonnington and Kuehn, 2015).

*Gardnerella vaginalis* is an anaerobic bacterium which grows well in less acidic pH (>4.5) of the vaginal milieu during BV, a widely prevalent genital tract infection (Aldunate et al., 2015). However, a healthy vagina characterized by low pH (3.5–4.5) may also harbor *G. vaginalis*, although in reduced numbers (O’Hanlon et al., 2013; Shipitsyna et al., 2013). A significant drop in CFUs of *G. vaginalis* indicates considerable extent of cell death upon exposure to low pH which mimics the physiological environment of a healthy vagina. This has been reported previously (Gottschick et al., 2016) and is also supported by our own results. Further, the reduction in viability of the cells exposed to low pH appears to be accompanied with a reduction in integrity of the proteins or an increase in protein degradation suggested by the qualitative changes in the SDS-PAGE profiles of total cellular proteins from cells grown at pH 3.5 compared to those grown at pH 6.5. Despite the substantial loss of cell viability observed due to acidic stress, there exists a possibility that some of the bacterial cells may survive and adapt to growth in low pH conditions (Aertsen and Michiels, 2004). However, this needs to be still experimentally validated. Nevertheless, the reduction in total protein as well as reduced expression of certain proteins over time could be indicative of both cell death and dormancy. The altered morphology of *G. vaginalis* exposed to pH stress characterized by increased vesiculation therefore reflects altered biogenesis of MVs in cells poised either for lysis or adaptation (Turnbull et al., 2016; Klimentova et al., 2019). The increase in MV production observed in response to pH stress appears to be similar to that reported in case of other stress conditions including stress induced by antibiotic treatment (Devos et al., 2015; Volgers et al., 2018). It is likely that a fraction of the stressed cells may undergo fragmentation followed by “self-annealing” of the fragments leading to generation of larger MVs (Turnbull et al., 2016) while the remaining cells adapt to the stress by actively producing smaller MVs in a regulated manner. This is also supported by the wider size distribution of MVs including both small and large MVs obtained in response to pH stress and demonstrated by DLS measurements which further hint toward altered biogenesis of MVs.

Acidic stress led not only to an increase in the release of MVs but also led to change in the protein integrity, i.e., increased protein degradation, also reflected by the diffused or smear pattern of the SDS-PAGE profile. The fact that MVs isolated from cells grown under non-stressed conditions (pH 6.5) do not exhibit a diffused protein profile upon exposure to acidic conditions post production indicates that the protein degradation observed in MVs isolated from cells grown under acidic stress at pH 3.5 is due to degradation of proteins in the cells from which they have been derived and not due to protein degradation in MVs post production and release from cells. Acidic stress also resulted in reduced protein content of the released MVs, demonstrated by flow cytometry. Qualitative analysis indicated that despite the apparent increase in number of MVs in response to pH stress, there was an overall reduction in protein content of these MVs. Quantitative flow cytometry analysis using dual florescence labeling for proteins and lipids revealed that although pH stress results in an alteration in release of MVs, there is an increase in the percentage of larger MVs (∼0.5 μm) along with a reduction in smaller MVs. It is likely that the increase in release of larger MVs is contributed by both cell death as well as cellular adaptation to stress. Further, there appeared to exist two distinct sub populations of these larger MVs arising most likely due to non-uniform protein distribution as a response to stress compared to a population of MVs without stress (pH 6.5). It is not clear whether pH stress results in either dysregulation of protein packaging to MVs or in selective protein packaging to a distinct subset of MVs.

It is known that stress conditions (such as pH stress) lead to a change in bacterial cell physiology (Baatout et al., 2007). This is reflected by an altered protein composition and may help the cell to survive in an environment characterized by reduced macromolecular stability and increased cellular damage. The protein composition of MVs is altered in response to pH stress with a drastic reduction in the number of proteins compared to non-stressed conditions. The presence of chaperones like Dnak and GroEL as well as the DNA binding protein-HB1 despite the overall reduction in protein diversity suggests that they could play an important role in acid tolerance by prevention of protein and nucleic acid denaturation (Susin et al., 2006; Henderson and Martin, 2011). It is evident that the presence of some of the proteins required for translation and carbohydrate metabolism, i.e., glycolysis and pentose phosphate pathway in the MVs is on account of their essential role in the survival of the bacterial cell. It is likely that some of the bacterial cells which have adapted to the pH stress may transfer these proteins via the MVs to rescue surrounding bacterial cells (Schwechheimer et al., 2014). It is notable that some of the protein constituents of the MVs produced under non-stressed conditions, e.g., proteins suggested to be involved in host cell invasion and virulence (Shishpal et al., 2020) including the pore forming toxin, vaginolysin are missing in MVs produced in response to pH stress.

Additionally, the absence of these proteins in the stress induced MVs could explain the apparent reduction of their ability to be internalized by the host cells as well as their diminished cytotoxic potential. Despite the reduced internalization and cytotoxicity of the vaginal epithelial cells, acidic stress induced MVs resulted in induction of the pro-inflammatory cytokine, IL-8. A likely explanation for this effect could be the presence of cell membrane and cell wall constituents such as lipopolysaccharide and peptidoglycan which may also be associated with these MVs (Pivarcsi et al., 2005).

This study for the first time provides an insight into the probable mechanisms for adaptation to acidic stress employed by *G. vaginalis*, an anaerobe usually associated with BV but also found in the vaginal tract of healthy women. The results of the study demonstrate that acidic stress encountered by *G. vaginalis* on account of exposure to low pH leads to modulation of the biogenesis of MVs. This is also reflected in the altered morphology of the bacterial cells as well as altered size distribution and protein content and composition as well as reduced cytotoxic potential of the stress induced MVs. Apart from being required for survival of *G. vaginalis* in the vaginal lactobacilli dominated, lactic acid induced, low pH environment of a healthy vagina, the modulation of MV composition and function is also in concordance with host physiology in absence of vaginal infection. The relevance of these findings could be further validated by comparing the compositional and functional profile of MVs obtained from vaginal tract of healthy women and women with BV. Though the present study focused on potential mechanisms of stress adaptation by *G. vaginalis* ATCC 14019, similar approaches could be used to investigate the phenomenon of adaptation to low pH by other anaerobes found, in the healthy vagina.

## Data Availability Statement

The original contributions presented in the study are included in the article/Supplementary Material; further inquiries can be directed to the corresponding author/s.

## Author Contributions

PS designed and performed the experiments, analyzed the results, and prepared the figures. VP provided vital support for design and interpretation of the flow cytometry experiments. DS provided support for performing electron microscopy experiments and also captured electron microscopy images. VB conceived the study, designed the experiments, interpreted the data, and critically reviewed the manuscript for intellectual content. VB and PS wrote the manuscript. All authors have read and approved the final manuscript.

## Conflict of Interest

The authors declare that the research was conducted in the absence of any commercial or financial relationships that could be construed as a potential conflict of interest.

## Publisher’s Note

All claims expressed in this article are solely those of the authors and do not necessarily represent those of their affiliated organizations, or those of the publisher, the editors and the reviewers. Any product that may be evaluated in this article, or claim that may be made by its manufacturer, is not guaranteed or endorsed by the publisher.

## Footnotes

1
www.uniprot.org
