# Supplementary material for: pH Stress Mediated Alteration in Protein Composition and Reduction in Cytotoxic Potential of Gardnerella vaginalis Membrane Vesicles
Source: Front Microbiol. 2021 Nov 2;12:723909. doi: 10.3389/fmicb.2021.723909 (PMC8593039; doi:10.3389/fmicb.2021.723909)
Supplement: Supplementary file 1 [file Data_Sheet_1.docx]

**pH stress mediated alteration in protein composition and reduction in cytotoxic potential of *Gardnerella vaginalis* membrane vesicles**

Parul Shishpal^1^, Vainav Patel^2^, Dipty Singh^3^ and Vikrant M Bhor^1*^

Affiliations:

^1^Department of Molecular Immunology and Microbiology,

^2^Department of Biochemistry,

^3^Department of Neuroendocrinology and Transmission Electron Microscopy,

Indian Council of Medical Research-National Institute for Research in Reproductive Health (ICMR-NIRRH), J. M. Street, Parel, Mumbai, India.

*Correspondence:

Dr. Vikrant M Bhor, Department of Molecular Immunology and Microbiology,

ICMR-NIRRH, J. M. Street, Parel, Mumbai, India. Email: [bhorv@nirrh.res.in](mailto:bhorv@nirrh.res.in),

Tel: +91-22-24192016, Fax: +91-22-24139412.

Running title: pH stress induced *G. vaginalis* membrane vesicles

**Supplementary Information**

**Supplementary Table 1: Size distribution of *G. vaginalis* MVs at pH 6.5 using Dynamic Light Scattering.**

| Size (d. nm) | Intensity (%) |
| --- | --- |
| 122.4 | 1.3 |
| 141.8 | 12.8 |
| 164.2 | 26.3 |
| 190.1 | 30.5 |
| 220.2 | 21.8 |
| 255.0 | 7.5 |

**Supplementary Table 2: Size distribution of *G. vaginalis* MVs at pH 3.5 using Dynamic Light Scattering.**

| Size (d. nm) | Intensity (%) |
| --- | --- |
| 190.1 | 2.5 |
| 220.2 | 8.0 |
| 255.0 | 14.2 |
| 295.3 | 18.6 |
| 342.0 | 19.6 |
| 396.1 | 17.0 |
| 458.7 | 11.9 |
| 531.2 | 6.3 |
| 615.1 | 1.9 |

**Supplementary Table 3: Percentage of dual stained (i.e. protein and lipid) MVs of varying sizes at pH 6.5 and pH 3.5**

| Size of MVs (µm) | pH 6.5  (Mean ± SD) | pH 3.5  (Mean ± SD)* |
| --- | --- | --- |
| 0.1 | 4.32 ± 3.5 | 4.6 ± 7.1 |
| 0.2 | 24.4 ± 23 | 11.6 ± 12.2 |
| 0.5 | 60.93 ± 30.2 | 79.6 ±15.1 |

***No statistical significance was observed between the percentage of MVs (0.1, 0.2 and 0.5 µm) obtained at pH 6.5 and pH 3.5.**
