# Supplementary figures and images for: pH Stress Mediated Alteration in Protein Composition and Reduction in Cytotoxic Potential of Gardnerella vaginalis Membrane Vesicles
Source: Front Microbiol. 2021 Nov 2;12:723909. doi: 10.3389/fmicb.2021.723909 (PMC8593039; doi:10.3389/fmicb.2021.723909)

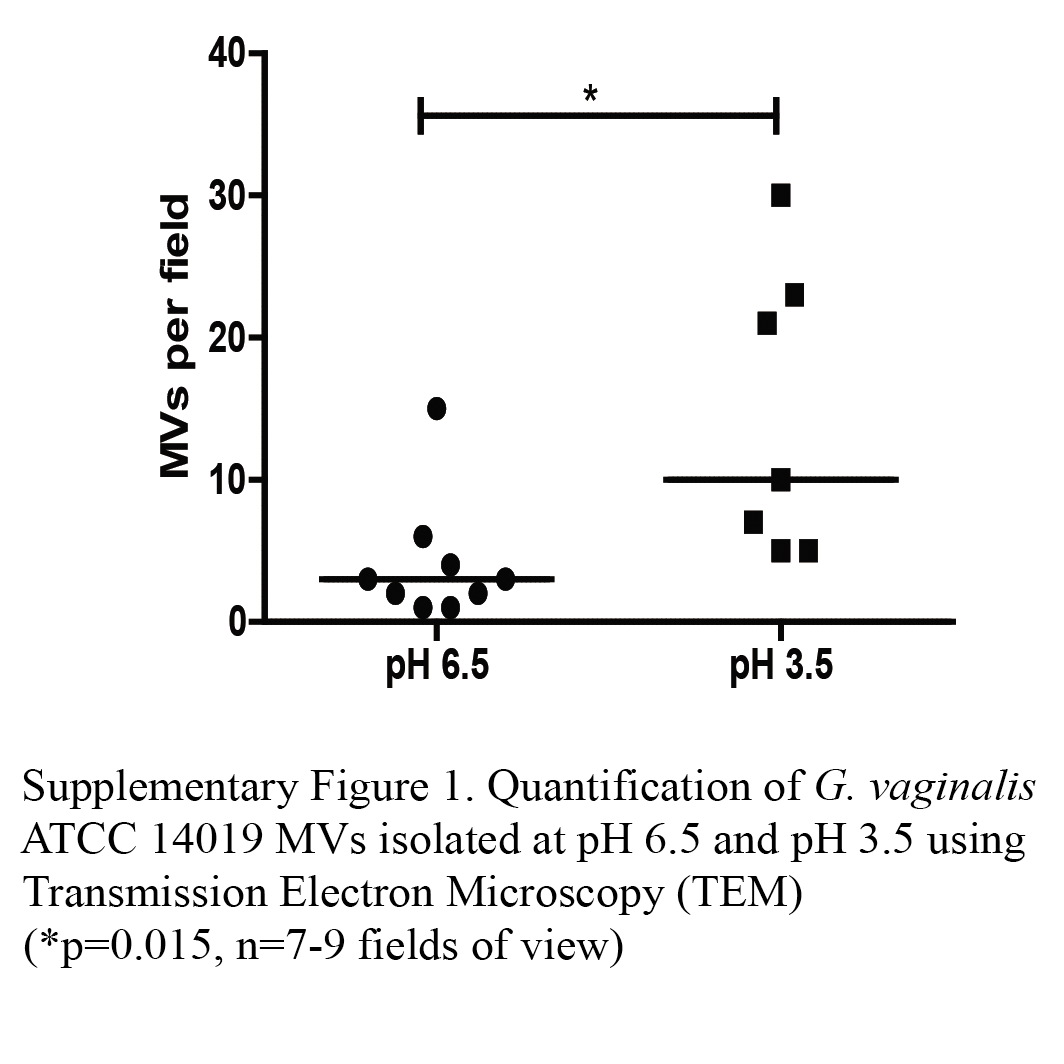

Supplement: Supplementary file 2 [file Image_1.tif]

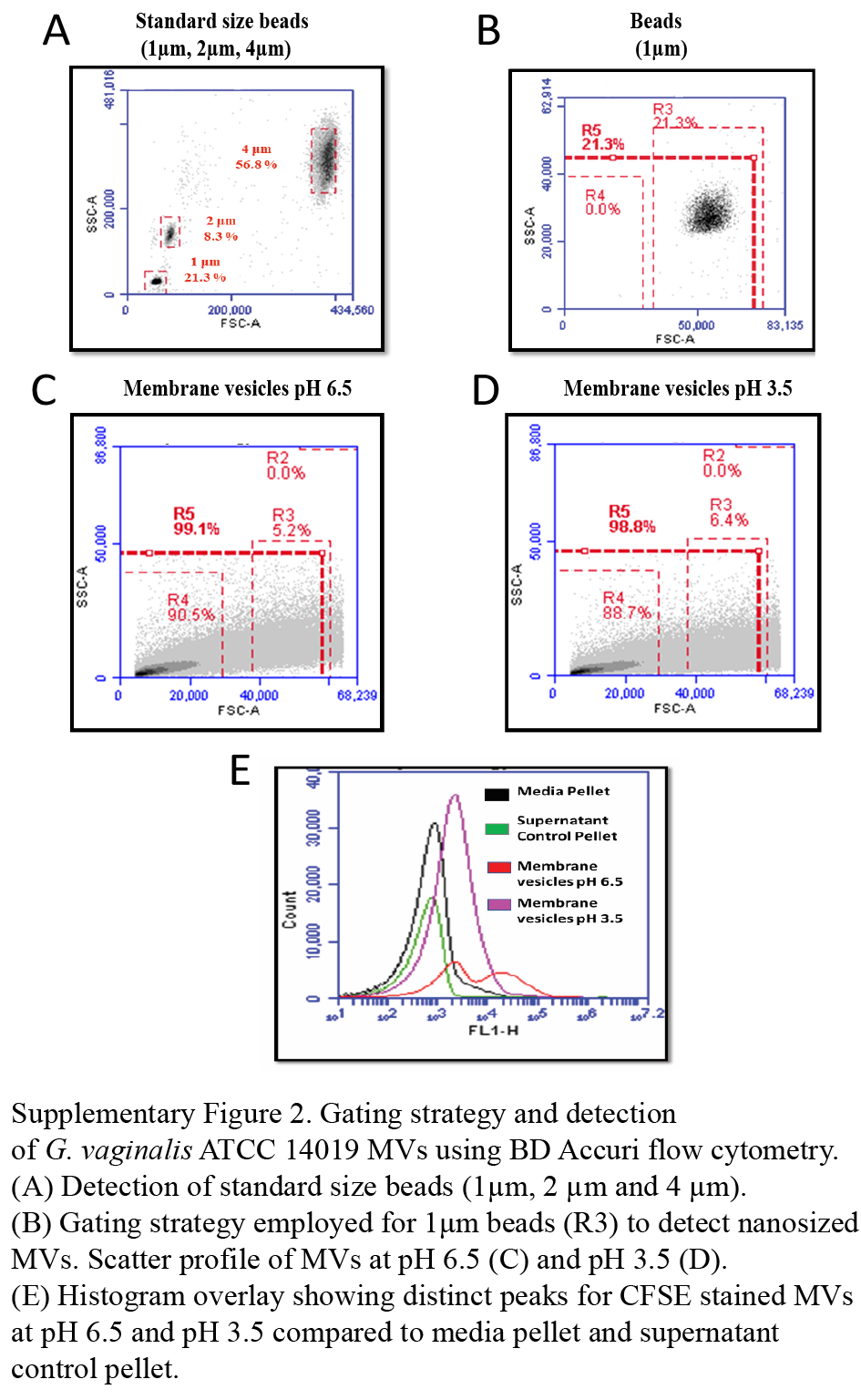

Supplement: Supplementary file 3 [file Image_2.tif]

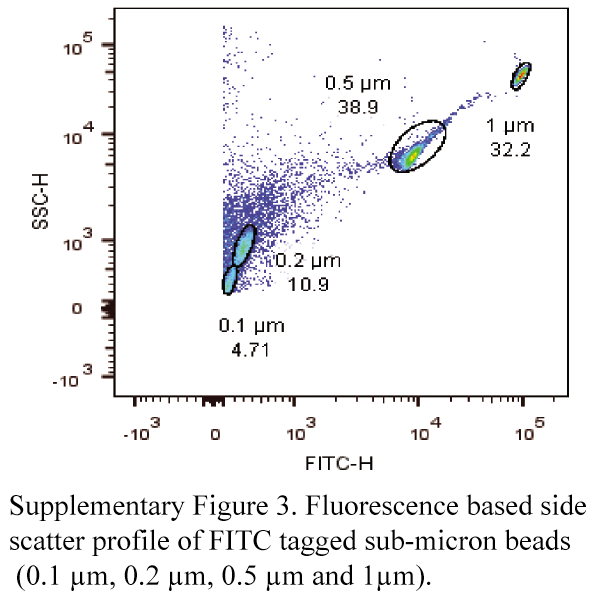

Supplement: Supplementary file 4 [file Image_3.tif]

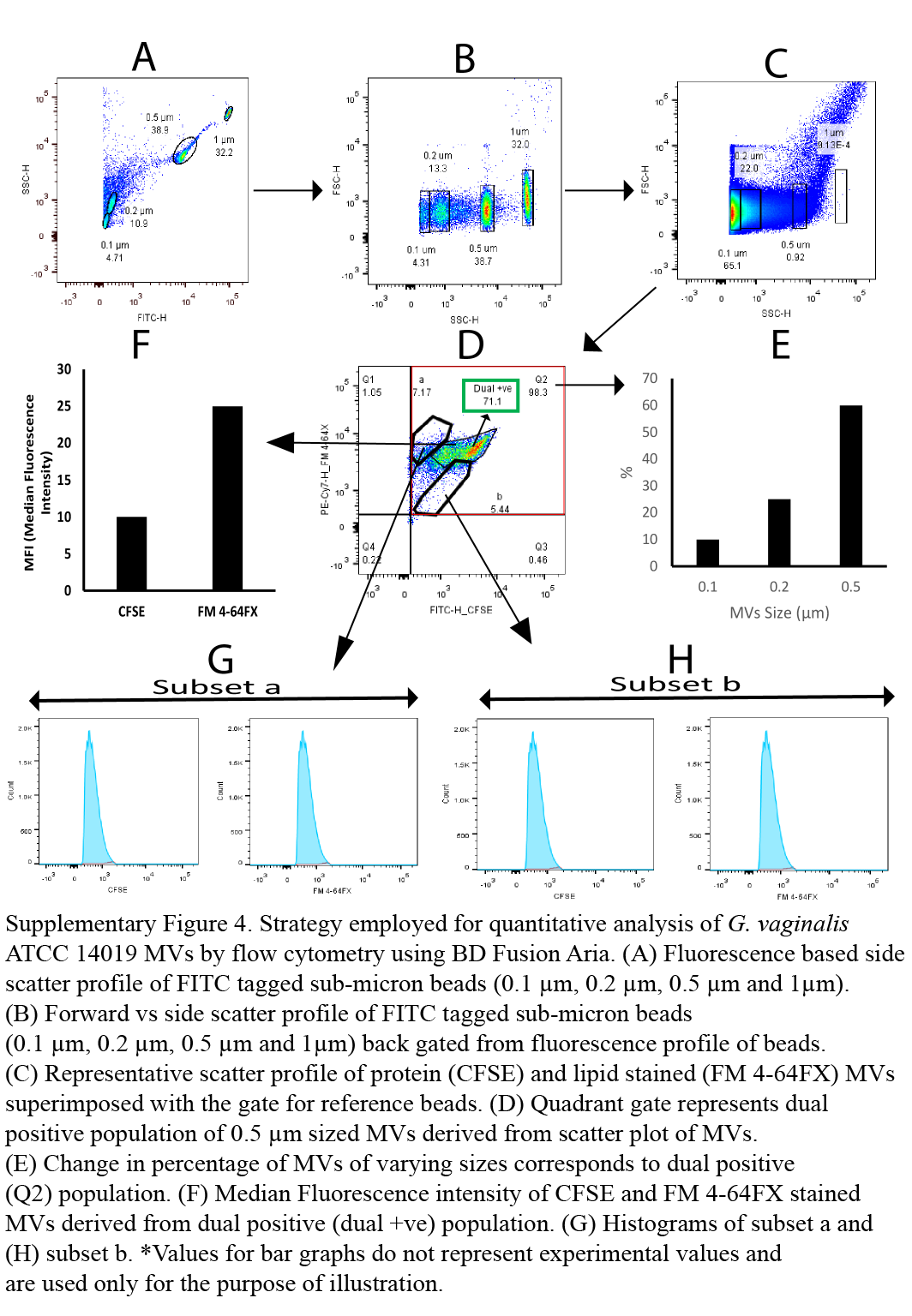

Supplement: Supplementary file 5 [file Image_4.tif]
